# The role of feed-forward and feedback processes for closed-loop prosthesis control

**DOI:** 10.1186/1743-0003-8-60

**Published:** 2011-10-27

**Authors:** Ian Saunders, Sethu Vijayakumar

**Affiliations:** 1 Institute of Perception, Action and Behaviour, School of Informatics, University of Edinburgh, UK

## Abstract

**Background:**

It is widely believed that both feed-forward and feed-back mechanisms are required for successful object manipulation. Open-loop upper-limb prosthesis wearers receive no tactile feedback, which may be the cause of their limited dexterity and compromised grip force control. In this paper we ask whether observed prosthesis control impairments are due to lack of feedback or due to inadequate feed-forward control.

**Methods:**

Healthy subjects were fitted with a closed-loop robotic hand and instructed to grasp and lift objects of different weights as we recorded trajectories and force profiles. We conducted three experiments under different feed-forward and feed-back configurations to elucidate the role of tactile feedback (i) in ideal conditions, (ii) under sensory deprivation, and (iii) under feed-forward uncertainty.

**Results:**

(i) We found that subjects formed economical grasps in ideal conditions. (ii) To our surprise, this ability was preserved even when visual and tactile feedback were removed. (iii) When we introduced uncertainty into the hand controller performance degraded significantly in the absence of either visual or tactile feedback. Greatest performance was achieved when both sources of feedback were present.

**Conclusions:**

We have introduced a novel method to understand the cognitive processes underlying grasping and lifting. We have shown quantitatively that tactile feedback can significantly improve performance in the presence of feed-forward uncertainty. However, our results indicate that feed-forward and feed-back mechanisms serve complementary roles, suggesting that to improve on the state-of-the-art in prosthetic hands we must develop prostheses that empower users to correct for the inevitable uncertainty in their feed-forward control.

## Background

For many decades researchers have considered the possibility of 'closing the loop' for upper-limb prosthesis wearers. Historically, feedback has been added to increase patient confidence [1] and to improve object grasping and lifting [2,3]. In the future we may see prosthetic hands that integrate directly with the amputee's nervous system, utilising state-of-the-art sensor technology [4,5] and relying on pioneering medical procedures [6-8]. Nevertheless, state-of-the-art upper limb prostheses are still open-loop devices with limited degrees of control, described as "clumsy" [9] and requiring considerable mental effort [10]. As technology continues to advance it is more important than ever that we find effective ways of delivering feedback to amputees.

Artificial feedback systems can exploit the idea of *sensory substitution*: feedback delivered in a different modality or to a different location on the body in an attempt to exploit the latent plasticity of the nervous system. For example, Multiple Sclerosis patients significantly over-grip objects [11], but when sufferers receive vibratory feedback of their grip force (displaced to their less-affected hand) these forces reduce [12]. In a similar way, prosthesis fingertip forces have been transferred to the stump [13] or even the toes of amputees [14] to create appropriate and useful sensations. Successful substitution is achieved when subjects no longer perceive the stimulation as an abstract signal but instead as an extension of their sense of touch. Achieving 'embodiment' in this sense depends critically on the presence of feedback [15]. Despite these promising results, few studies have objectively quantified the benefits of artificial tactile feedback. One must not only question the efficacy of the feedback method (e.g. its resolution and latency) but also identify what feedback information should be provided and observe how well it integrates with our existing sensory processes (i.e. whether their presence obviates its utility [16]). A key feature of human grip force control is the ability to act in a *feedforward *manner, a mechanism by which people act in anticipation of their actions in the absence of *externally-arising *cues. The formation and maintenance of internal models has been studied in healthy individuals (reviewed in [17]), but the coupling between feedforward and feedback processes has not been studied in prosthesis wearers.

Research in intact and deafferented humans has suggested that both feedback and feedforward mechanisms are required for successful object manipulation, with a marked disassociation between these aspects of control [18]. The difference between feedforward and feedback processes is of fundamental importance to our understanding of human sensorimotor behaviour [19], and likewise should be considered crucial in designing a prosthesis to improve the quality of life for amputees. Feedforward anticipatory grip forces precede load changes due to acceleration, a phenomenon unimpaired by digital anaesthesia [20] and long-term peripheral sensory neuropathy [21]. In contrast, the scaling of grip force magnitude is not preserved under anaesthesia, resulting in over-grip and unstable forces [20], suggesting that cutaneous cues are required to allow us to maintain our forward model of grip force. These studies indicate a vital role of tactile feedback for both learning and maintenance of internal models.

**Figure 1:**
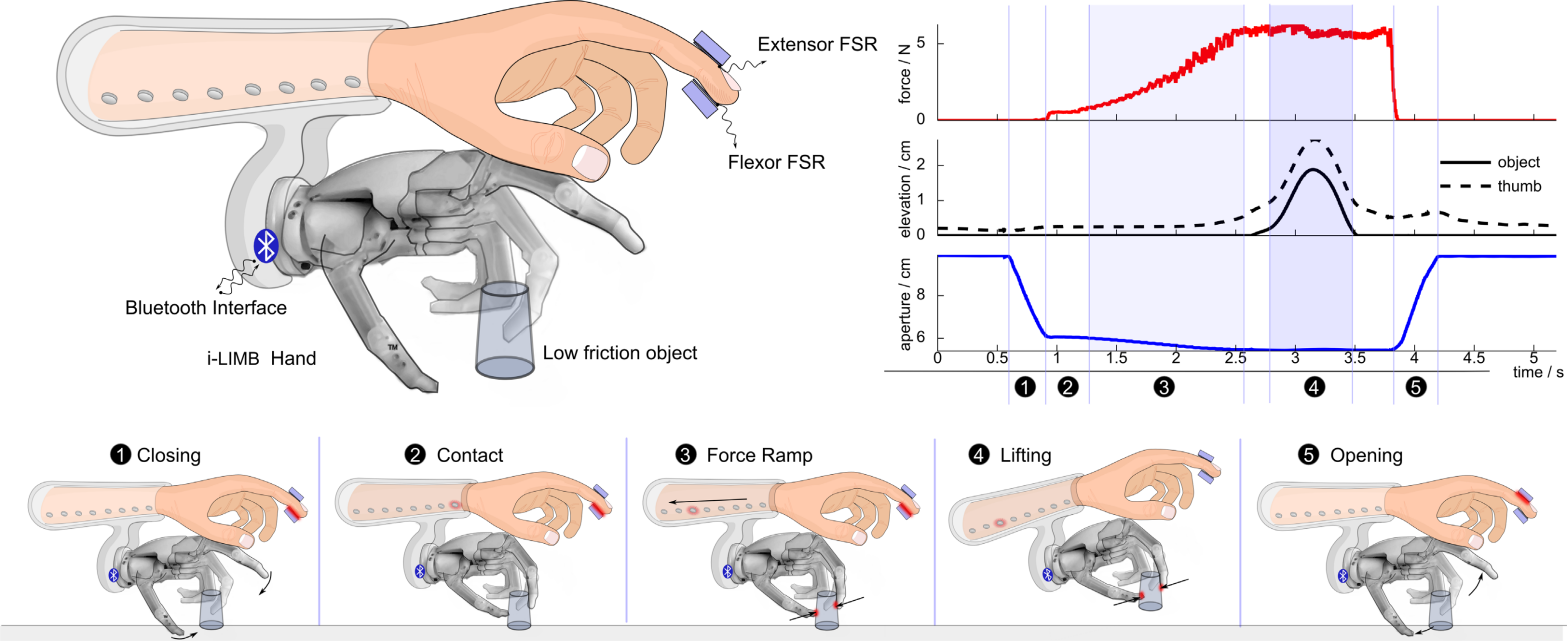
**The 'Grasp and Lift' paradigm with our Closed-Loop prosthetic hand**. Healthy subjects were fitted with a modified i-limb Pulse prosthetic hand with a two-channel differential force controller. Grip-force feedback was delivered to their arm using a vibrotactile feedback array (see *methods*). They were instructed to grasp, lift and replace a low-friction object (inset 1-5). A typical trajectory (showing grip force, object and thumb elevation, and grasp aperture) is also shown.

In this study we use the behavioural phenomenon of *economical grasping and lifting *to quantify the contributions of these fundamental processes in prosthesis control. Economical grasping is a stereotypical human behaviour in which grip forces scale appropriately with objects of different loads (minimising effort yet avoiding slip). This phenomenon has been characterised for both healthy [22] and sensory-impaired subjects [20,21]. In this study we augment healthy subjects with an artificial extension to their nervous system (Figure 1), creating a model system in which we can readily manipulate the control interface, the robotic controller, on-board sensors, and feedback transduction. Using this closed-loop manipulandum we observe the effect of artificial sensory impairments on the phenomenon of grasping and lifting.

We conducted three experiments designed to focus specifically on the interaction between feedforward and feedback processes. In our first experiment we created an idealised scenario in which sensory and motor uncertainty were minimised. Subjects were asked to grasp, lift and move an object, and we provided vibrotactile force feedback on 50% of the trials. We hypothesised that under 'simulated anaesthesia' subjects would still be able to grip economically, albeit with larger variability and more errors, since anaesthesia does not impair anticipatory force control in healthy individuals [20]. In our second experiment we deprived subjects of visual, tactile and auditory feedback in order to quantify the resulting benefits of vibrotactile feedback in the absence of all other sensory cues. Intermittent sensory feedback is necessary to update and maintain internal models of object dynamics [18] and vibrotactile feedback has been shown to be beneficial under partial sensory deprivation [16]. We therefore hypothesised that under complete sensory deprivation economical grasping ability would decline, but in the presence of vibrotactile feedback it would not. An unexpected result in the second experiment suggested that another strategy was employed in the absence of feedback, sufficient for subjects to negotiate an efficient grip force. We hypothesised that this may be due to feedforward information and sought evidence for this hypothesis through our third experiment. We induced temporal unpredictability to the controller in order to manipulate feedforward uncertainty to quantify the utility of visual and vibrotactile feedback under feedforward uncertainty. By adding temporal unpredictability to the hand, subjects experience reduced utility of feedforward control. We hypothesised that this would increase their dependency on vibrotactile feedback. Together these experiments provide a window into the role of feedforward and feedback processes for prosthesis control. In this study we aim to explore a well characterised behavioural phenomenon using a novel sensorimotor platform, open to arbitrary manipulation. Our results confirm differential roles for feedforward and feedback processes, and reveals their complementary nature.

## Methods

### Subjects

Subjects were healthy males and females, all right-handed and aged between 21 and 30 years old, sampled from the academic institute in which the research was conducted. They had both upper limbs intact, and had normal or corrected-to-normal eyesight. None of the subjects had previous experience controlling a prosthesis.

The experimental protocols were in compliance with the Helsinki Declaration and assessed in accordance with the University of Edinburgh School of Informatics policy statement on the use of humans in experiments, approved by the Planning and Resources Committee and the Research Advisory Committee. All subjects gave informed consent before participation in the study.

### Hardware Setup

#### Closed Loop Hand

Healthy subjects were fitted with a modified Touch Bionics i-limb Pulse prosthetic hand on their dominant (right) hand (Touch Emas, UK), using a custom-built 'socket' (Figure 1). This state-of-the-art, commercially available prosthesis has a differential (open/close) controller, driven by two surface electromyography (EMG) electrodes. The hand has 5 individually-powered digits, and a bluetooth interface to allow real-time streaming of data to a PC for data logging. It has scored highly in terms of patient satisfaction [23] and is an open-loop hand, making it an ideal candidate for developing a feedback system. We modified the firmware of the hand to enable differential force control.

#### Differential Force Control

We used a 'gated ramp controller, for two-channel differential position and force control (e.g. see [24]). Subjects controlled the hand using extensor and flexor signals detected by force-sensing resistors (FSRs) rigidly attached to the fingertip (see Figure 1). For simplicity of operation, the signals operated as binary switches. The flexor signal closed the hand at a constant speed of 0.12m/s, and when contact was made the force ramped up at approximately 5N/s. The extensor signal opened the hand at a constant speed of 0.12m/s. This simple controller allowed subjects to control the force they exerted, in the range 0-15N, by modulating the duration of the signal. We chose this method as it is similar to the existing controller on the i-limb pulse hand, which is a highly successful open-loop prosthesis.

#### Vibrotactile Feedback

A 'vibrotactile feedback array' was constructed using eight 10 mm diameter shaftless button-type vibration motors (Precision Microdrives, UK). These were each connected to transistors on the output of digital latches, to enable the switching on and off of each motor when the appropriate digital signal was sent from a PIC18F4550 microcontroller (Microchip, USA). The microcontroller was running custom firmware, including a universal serial bus (USB) module that enabled a personal computer (PC) to control the vibrotactile stimulation. The hardware allows us to control the pulse width and period of stimulation. This enabled independent control of the duty cycle and frequency of pulses to each motor. Our firmware modulation allowed motor patterns at frequencies ranging from 2 Hz to 200 Hz, and with pulse-widths of 500*μ*s to 64 ms.

Subjects were fitted with a socket containing the vibrating motors (shown in Figure 1). The eight motors spanned the full length of the palmar-side of the forearm. The grip force on the object was translated into a stimulation location: weak forces were perceived near the wrist and string forces (up to 10 N) near the elbow. To further increase the resolution of this tactile display we devised a method to create 'between-motor' sensations, achieved by co-stimulation of neighbouring motors.

#### Sensor Recording Equipment

A large FSR (5 cm square) was attached to the object being lifted. The sensor was calibrated using high precision digital scales, so that the force output could be accurately recorded at 1 kHz in the range 0N to 10N, using a 10-bit analogue-to-digital converter (ADC) on the the microcontroller, streamed to PC software. Position sensors were attached to the thumb and forefinger, the wrist and the base of the object, to enable accurate three dimensional tracking using a Polhemus Liberty 240 Hz 8-sensor motion tracking system (Polhemus, USA), and logged by PC software. The i-limb hand was configured to stream state information, such as control signals from the EMG inputs to the hand, via bluetooth to the PC software.

All data were collated using the same PC software to ensure accurate temporal calibration. Force feedback was streamed back to the microcontroller for provision of vibrotactile feedback.

### Experiments

#### Preliminary Experiment: 'Just noticeable difference' measurement

To establish the efficacy of the feedback system, we ran an adaptive-staircase design two-interval forced-choice protocol. Subjects (N = 6) were presented with two successive vibrotactile stimuli (10 ms duration, 3 ms separation) and asked to report if the second stimulus was located to the right or to the left of the first. This was done at 6 reference locations along the forearm. Probe stimuli locations were chosen, as per the adaptive-staircase design, to converge on the 75% just-noticeable-difference (JND) threshold. This is the threshold at which subjects correctly determine the location on 75% of the trials, where 'chance' is at 50%. Subjects received 20 pairs of stimuli for each location, which was sufficient to establish a per-subject psychometric curve and a per-location psychometric curve (across subjects).

#### Overview: Economical Grasping Paradigm

Healthy individuals exhibit stereotypical and repeatable grasping profiles [22,25] and the term 'economical grasp' describes this ability to minimise grip force while avoiding slip. This phenomenon relies on both feedforward and feedback mechanisms (see *introduction*).

In our three main experiments, subjects were given on-screen instructions to grasp and lift objects with sufficient force, and to avoid dropping or over-gripping the object. Two objects were used, one 'heavy', (300 g) and one 'lightweight' (150 g). The objects were upward-tapered identical rigid beakers, 55 mm diameter at the point of contact, covered with a low-friction cellulose film. Since we are primarily interested in establishing whether or not subjects are able to differentially control their grip force, we define an economical grasp occurring when subjects are able to appropriately assign different grip forces to the two objects (Note: in the third experiment we use just the heavy object to reduce the experiment complexity, and so ability at this task is judged by the difference in measured performance magnitude between the feedback conditions.)

#### Experiment 1: Grasp, lift and move task

**Figure 2:**
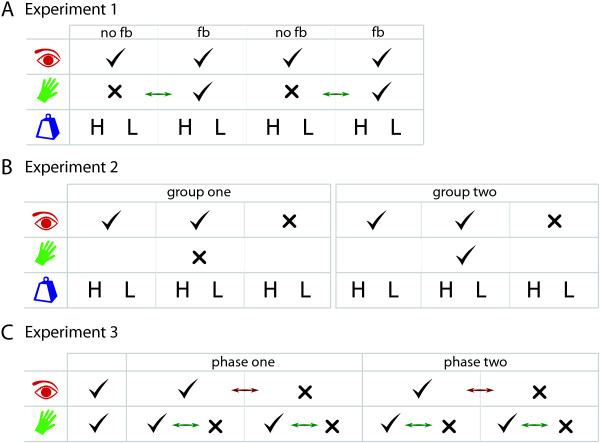
**Experiment Overview**. We conducted three behavioural experiments to examine the role of feedback. **(A) **In Experiment 1 we allowed subjects to use visual feedback throughout, and alternated the presence of vibrotactile feedback. Object weight (lightweight, 'L', and heavy, 'H') varied between blocks as shown. The order of presentation of feedback was counterbalanced (indicated by the double-headed arrow). **(B) **In Experiment 2 we used two groups of subjects, one with vibrotactile feedback and one without. Subjects performed two blocks with visual feedback, and a third immersed in darkness, with different object weights. **(C) **In Experiment 3 subjects had an initial training phase, then had two phases of trials in all four feedback configurations (visual, tactile, neither and both), counterbalanced as shown.

In our first main experiment we intended to create idealised conditions. The i-limb hand was controlled using FSRs, so that it would respond immediately and predictably to control signals. Subjects were allowed to use visual feedback throughout, and performed repeated trials with each object weight. Subjects (N = 6) were fitted with the i-limb socket with vibrotactile motors along the palmar forearm. On a given *trial *subjects were instructed to grasp, lift and transfer an object between two locations, spaced 20 cm apart. After each trial subjects received on-screen feedback of their peak grip force during the trial. Subjects performed four *blocks *of trials, each of which included 20 trials with the heavy object and 20 trials with the lightweight object. In a given block, each subject was exposed to one of two counterbalanced experimental conditions: either *with *or *without *vibrotactile feedback of grasp force (see Figure 2). In our analyses we examined the effect of *tactile feedback condition *and *object weight *on performance.

#### Experiment 2: Grasp and lift task with feedback deprivation

In our second main experiment we examined performance when subjects were deprived of all useful sources of feedback: visual, auditory and additional tactile cues were eliminated. We compared two groups under this sensory deprivation condition so as to observe the benefit of tactile feedback alone on performance. Twelve subjects were split into two groups for *vibrotactile feedback condition*. One group (N = 6) had vibrotactile feedback for the duration of the experiment, and the other group (N = 6) received random (uncorrelated) tactile stimuli.

On a given *trial*, subjects were instructed to grasp and lift an object in a fixed location, then return it to the same location. After each trial subjects received on-screen feedback of their peak grip force during the trial. Subjects experienced three *blocks *of trials, two in the light, and one in the dark. Each block included 12 trials with a heavy object and 12 trials with a lightweight object.

Visual feedback was removed by immersing subjects in darkness. The robotic hand and the object were covered in dark materials so that the hand and its movements were not visible at any time. Subjects were also instructed to look at a screen throughout the trial, though they were able to see if the object had been successfully lifted by observing the movement of a phosphorescent strip attached to the top of the object. Auditory feedback was removed by playing white noise through earphones, and separately through a speaker. Additional sources of tactile feedback, such as vibrations when contact is made or during force ramping, were removed by the use of random (uncorrelated) vibrotactile stimuli. These stimuli appeared at random locations on the arm, vibrating with randomised frequencies and for unpredictable durations. In our analyses we examined the effect of *tactile feedback condition*, *visual feedback condition *(block 2 versus 3), and *object weight *on task performance.

#### Experiment 3: Grasp and lift task with feedback deprivation and feedforward deprivation

In our third main experiment we added feedforward uncertainty by inducing random unpredictable delays to the hand controller. In contrast to experiments 1 and 2, where the control of the hand was repeatable and predictable, this experiment was designed to examine the role of feedback under motor uncertainty, such as is more typical in real-world situations. We added random delays to the hand motion before the onset of movement and before the onset of the force ramp. Delays were drawn uniformly from the interval 0 s to 1.5 s, the order of magnitude of a typical hand movement, simulating the grasping of unknown-size objects (see *discussion*). Each subject (N = 12) was exposed to four different feedback conditions. We modified both the *visual feedback condition *(light versus dark) and *tactile feedback condition *(vibrotactile feedback versus no feedback). For each condition subjects performed a block of 12 trials. In a given trial, subjects were instructed to grasp and lift an object in a fixed location, then return it to the same location, as per experiment 2.

We used a within-subject design to reduce the effects of inter-subject variability. Since using a within-subjects design it was important to minimise interaction between the order of blocks and subject's ability to control the hand. We therefore mixed the subjects into four between-subject *groups*. Each group had a different configuration of the *visual feedback order *and the *tactile feedback order*, to ensure any learning effects were counterbalanced. This enabled us to control for carry-over effects within-subjects. Furthermore, we also trained subjects briefly before the start of the first trial, with full feedback sensibility, so that they could get used to the control mechanism of the hand.

Subjects performed the four blocks of the experiment over two separate *phases*. This would allow us to detect any effects of learning across phases. We used the same object for all trials to simplify the design. In our analyses we examined the effect of *tactile feedback condition*, *visual feedback condition *and the *phase *of the experiment. We also ensured that there were no effects of *visual feedback order *or *tactile feedback order *which might confound the results. One subject was discarded from these analyses as he used a different strategy to complete the task (the subject was able to detect successful contact using his free hand).

### Performance measures and statistical analysis

#### Automatic Segmentation

Data from each trial were automatically segmented. Data were annotated to mark occasions where the object slipped or was dropped. We located the start and end of the force ramp, and the period for which the object was elevated. Figure 1 shows a typical recorded trajectory, and illustrates segmentation features. Phases 3 and 4, highlighted, are the 'force ramp' and 'lifting phase' respectively This temporal segmentation allows us to compute the duration of the motion, count the number of errors made, and compute the grasp force during object lift.

#### Grasp Force

A key indicator of economical grasping is avoidance of over-grip. Lightweight objects should be gripped with less force than heavier objects. For a given trial *i *we therefore define the grasp force, *f_i_*, as the average grip force (in Newtons) applied to the object for the duration of its elevation.

#### Ramp Duration

The duration of the control signal is directly related to the subjects intended grasp force. This is a more reliable indicator of force than the FSR reading, as subjects might make imperfect contact with the sensor. For a given trial *i *we define the ramp duration, *r_i_*, as the duration in milliseconds of the force ramp phase, excluding any random delays induced in experiment 3.

#### Trial Duration

For a given trial *i *we define the trial duration, *d_i_*, as the duration in milliseconds of the entire trial, excluding any random delays induced in experiment 3.

#### Number of errors

For a given trial *i *we define the number of errors, *e_i_*, as the sum of 'drops', 'slips' and 'failed lifts'. A drop occurs when the object is in a stable grasp (between the thumb and forefinger with grip force> 1 N), and the downward acceleration of the object is 5 m/s^2 ^greater than the downward acceleration of the thumb. A slip occurs when the object is in a stable grasp, and the upward velocity measured at the tip of the thumb is greater than the upward velocity measured at the base of the object by more than 0.05 m/s. A failed lift occurs when the object is not in a stable grasp (grip force< 1N) and the upward velocity measured at the tip of the thumb is greater than the upward velocity measured at the base of the object by 0.05 m/s. If two errors are detected in a given 60 ms period we count this as just one error.

#### Grasp Score

We devised a compound metric to handle inter-subject variability: a per-trial grasp score *s_i_*, rates each trajectory, *i*, in terms of both speed and accuracy. A higher grasp score indicates worse performance. This metric is comprised of four terms, to capture the grasp force, *f_i_*, the ramp duration, *r_i_*, the trial duration *d_i_*, and the number of errors, *e_i_*, defined as follows:

(1) $$s_{i}=\text{norm}\left(f,i\right)+\text{norm}\left(r,i\right)+\text{norm}\left(d,i\right)+e_{i}$$

(2) $$\text{norm}\left(x,i\right)=\frac{x_{i}-\text{target}\left(x\right)}{\text{peak}\left(x\right)-\text{target}\left(x\right)}$$

(3) $$\text{target}\left(x\right)={\text{min}}_{j}\left(x_{j}|e_{j}=0\right)$$

(4) $$\text{peak}\left(x\right)={\text{max}}_{j}\left(x_{j}\right)$$

target computes the best performance from a given subject's successful trials (i.e. only using trials in which there were no errors, denoted by the conditional term). This is therefore a measure of the subjects target performance. peak, is a measure of the subject's worst performance over all trials. norm uses the target and peak functions to normalise each trajectory into a *per-subject *range, where *s_i _*= 0 indicates good performance on trial *i*, and *s_i _*≥ 1 indicates bad performance on trial *i*.

### Analyses

In our subsequent data analyses we use the *grasp force*, *duration of ramp *and the *grasp score *measures to compare performance. In a pilot trial these were determined to be the most relevant measures of a successful grasp. We correct for the use of repeated measures in our statistical analyses (except where univariate results are explicitly reported).

## Results

### Preliminary Experiment: We can effectively communicate grasp forces to patients using artificial feedback

Before using our tactile feedback interface we conducted a preliminary experiment to verify that its efficacy (bandwidth) would be satisfactory to enable economical grasping. We calculated the just-noticeable-difference (JND) threshold of the stimuli using an adaptive-staircase forced-choice design (see *methods*). Data for all six subjects were combined.

**Figure 3:**
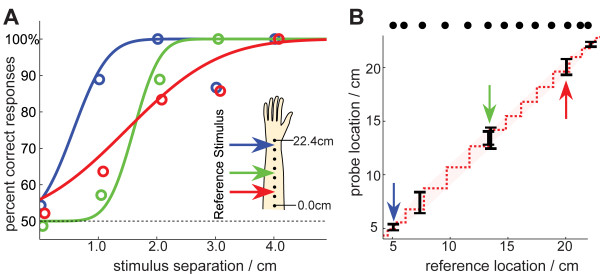
**Just Noticeable Difference (JND) experiment**. We measured subjects' ability to distinguish adjacent vibrotactile stimuli. Reference stimuli were chosen at six locations starting from the wrist (location 0) to the elbow (location 255). **(A) **Psychometric curves at three separate locations along the arm. The coloured circles correspond to average response data when binned into groups of 10 data points. The psychometric curves are Cumulative Gaussians fit to the raw data. **(B)**Sensitivity along the forearm can be plotted as a function of the success at distinguishing any two given stimuli. The 75% JND thresholds (black bars) suggest a region of stimulus indistinguishability (red shaded region). From this region we calculate the number of just-distinguishable stimuli, shown by the black blobs. This analysis indicates that approximately 12 distinguishable stimuli can be perceived along the forearm.

A cumulative Gaussian function was fitted to the proportion of correct responses as a function of stimulus separation. Figure 3A shows curve fits at three locations along the arm. As our adaptive staircase method does not give evenly distributed points, we do not fit the curve to binned data (though it is also shown for comparison). In Figure 3B we plot the across-subject JND threshold as a function of stimulus location. The results indicate that 12 discriminable levels are attainable over the length of the forearm, and sensitivity increases near the wrist and elbow.

### Experiment 1: In ideal conditions, subjects perform economical grasps regardless of feedback

In our first main experiment we measured grasp economy for prosthesis wearers under ideal conditions. *Economical grasping *is achieved when subjects appropriately assign different grip forces to objects of different weight (see *methods*).

To create *ideal conditions*, the robot hand was attached to healthy individuals and was controlled with a noise-free, predictable and responsive differential force-control algorithm (see *methods*). In a given block of trials subjects were asked to grasp, lift and move an object multiple times, with visual feedback throughout. Vibrotactile feedback was provided on some blocks (see *methods*).

**Figure 4:**
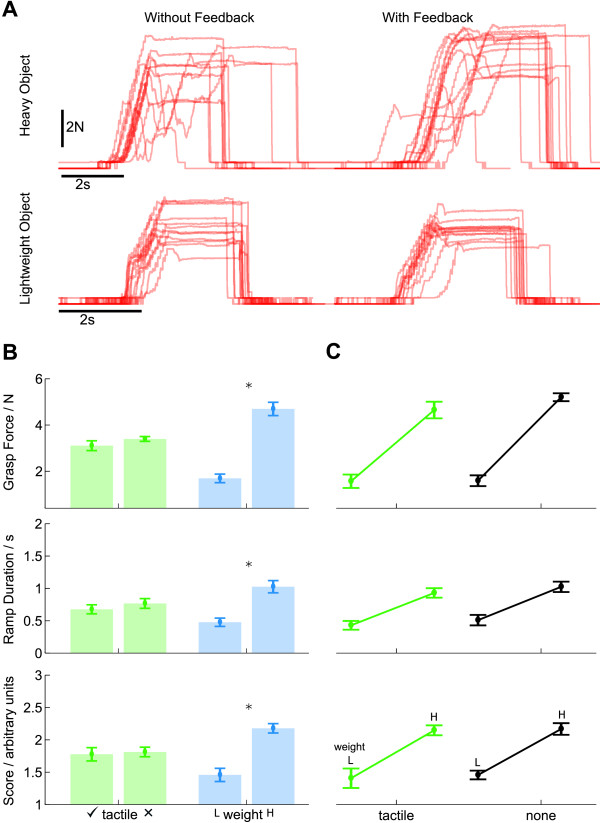
**Grouped results from Experiment 1**. **(A) **Sample grasp-force trajectories from Experiment 1, from a single subject. In each plot the x-axis denotes time in seconds, and the y-axis the force in Newtons. The plots show four different experimental conditions: lifting a heavy object without *(top left)*, and with vibrotactile feedback *(top right)*; lifting a lightweight object without *(bottom left)*, and with vibrotactile feedback *(bottom right)*. For this subject, tactile feedback offers little utility in reducing grasp force, only in reducing variability. Object weight, on the other hand, has a clear effect on grasp forces. **(B) **Data from Experiment 1, grouped by factor, using three metrics to compare performance. Error bars denote standard error. N = 6. Comparison of within-subject factors of tactile feedback condition (*green *bars) and object weight (*blue *bars). Weight is split into lightweight ('L') and heavy ('H'). ANOVA results revealed a significant main effect of object weight, but not of tactile feedback condition, denoted by the stars. **(C) **Data from Experiment 1, grouped by feedback condition, using three metrics. Error bars denote standard error. N = 6. Comparison of subjects' ability to discriminate object weight as a function of feedback condition. Feedback conditions were with tactile feedback ('tactile') and without tactile feedback ('none'). The two bars per condition indicate performance with the lightweight object ('L') and heavy object ('R'). Successful discrimination is indicated by a positive slope. Subjects were able to discriminate equally well in either feedback condition.

The force trajectories for one subject are shown in Figure 4. The data indicates that, for this subject, while there was less variability when vibrotactile feedback was available, economical grasps were formed regardless of feedback condition: the lightweight object is grasped with less force, and the heavier object with greater force. This phenomenon is consistent across subjects.

In order to evaluate this observation statistically, we reduced the recorded data to three measures of performance: *grasp force*, *duration of force ramp *and *grasp score *(see *methods*). Figure 4 shows the data grouped across subjects.

A within-subjects ANOVA, with factors of *object weight *(heavy/lightweight) and *tactile feedback condition *(with vibrotactile feedback/without vibrotactile feedback) revealed a significant main effect of *object weight *(*F*(3, 3) = 659, *p <*.001), but no significant effect of *tactile feedback condition *(*F*(3, 3) = 2.61, *p *= .226), and no interaction (*F*(3, 3) = 1.42, *p *= .390) The main effect of *object weight *was significant on all measures (*F*(1, 5) ≥ 92.9, *p *≤ .001). However, no significant effect of *tactile feedback condition *was found for any of the three measures (*F*(1, 5) ≥ 2.74, *p *≤ .159).

### Experiment 2: When deprived of additional sensory cues, trained subjects show no significant deficit in grasp economy

In our second main experiment we measured grasp economy for prosthesis wearers under ideal conditions with all additional sensory cues removed (visual, tactile and auditory, see *methods*). As a preliminary trial we observed a single naive subject in the dark (data not shown). We found that performance was greatly impaired in the initial dark block. Over all 10 trials the subject failed to supply enough force to successfully lift the object. However, the same subject completed the task with ease in a second dark block after 10 trials of vision-assisted training.

**Figure 5:**
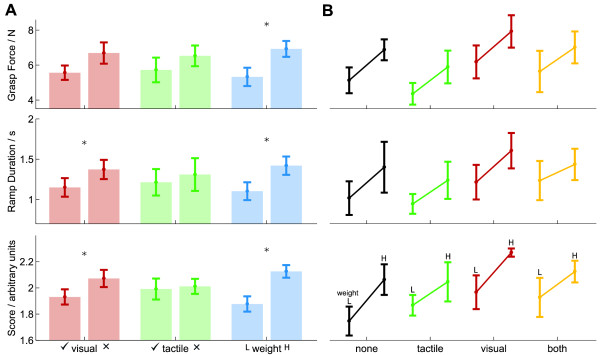
**Grouped results from Experiment 2**. Three metrics are used to compare performance. Error bars denote standard error. Data are from two groups of subjects, one with vibrotactile feedback (N = 6), one without vibrotactile feedback (N = 6). **(A) **Comparison of within-subject factors of visual feedback condition (*red *bars), tactile feedback condition (*green *bars), and object weight (*blue *bars). There was a significant within-subjects effect of both object weight and visual feedback condition, but not tactile feedback condition. Post-hoc results confirmed these differences (denoted by stars, significance at the *p *= .05 level.) **(B) **Comparison of subject's ability to discriminate object weight as a function of feedback condition. Feedback conditions were (*left to right*): no feedback; vibrotactile feedback only; visual feedback only; and both visual and tactile feedback. The two bars per condition indicate performance with the lightweight object (*left*) and heavy object (*right*). Successful discrimination is indicated by a positive slope. Subjects discriminated well in all feedback conditions, including in the absence of any feedback.

In a full experiment we compared performance with and without tactile feedback between two distinct groups. Subjects were exposed to three blocks of trials, the first two in the light and the third in the dark (see *methods*). The grouped data are shown in Figure 5. A between-subjects ANOVA, with factors of *object weight *(heavy object/lightweight object), *visual feedback condition *(light block/dark block) and *tactile feedback condition *(with vibrotactile feedback/without vibrotactile feedback) revealed a significant main effect of *visual feedback condition *(*F*(3, 8) = 4.68, *p *= .036). While no significant main effect was found for *object weight *(*F*(3, 8) = 2.1, *p *= .179), univariate tests did reveal a significant effect of *object weight*, on all three measures: *grasp force *(*F*(1, 10) = 7.84, *p *= .019), *ramp duration *(*F*(1, 10) = 5.01, *p *= .049) and *grasp score *(*F*(1, 10) = 6.58, *p *= .028). Univariate tests also confirmed the main effect of *visual feedback condition *(*F*(1, 10) ≥ 7.62, *p *≤ .020, all measures). There was no significant between-groups main effect of *tactile feedback condition *(*F*(3, 8) = 0.218, *p *= .881) and univariate tests also revealed no significant effect on any measure of performance of *tactile feedback condition *(*F*(1, 10) ≤ 0.764, *p *≥ .402).

### Experiment 3: When feedforward uncertainty is increased, trained subjects show significant performance deficits when deprived of either visual or tactile feedback

**Figure 6:**
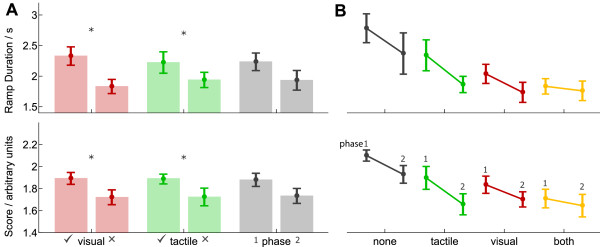
**Grouped results from Experiment 3**. Two metrics are used to compare performance. Error bars denote standard error. Data are from one cohort of subjects (N = 11). **(A) **Comparison of within-subject factors of visual feedback condition (*red *bars), tactile feedback condition (*green *bars), and trial phase (*grey *bars). Within-subjects ANOVA revealed significant main effects of visual feedback condition and tactile feedback condition, but not phase, indicated by stars. For detailed statistics see text. **(B) **Comparison of subjects' performance as a function of feedback condition: (*left to right*) no feedback; vibrotactile feedback only; visual feedback only; both visual and tactile feedback. The two bars per condition indicate performance in the first (*left*) and second (*right*) phases of training. Subjects performed significantly worse in the absence of either source of feedback.

Experiments 1 and 2 indicate that tactile feedback may offer limited practical utility for grasp force control if the hand controller is predictable. In the third main experiment we added uncertainty to the hand controller, in the form of brief randomised delays (see *methods*). This unpredictability was used to reduce subject's ability to form an accurate feedforward estimate (see *discussion*). The grouped data are shown in Figure 6.

In experiment 3 subjects found the task more difficult (indicated by a higher mean *grasp score *compared to experiment 2). Under the increased difficulty we found that subject's grasp forces were outside the linear range of our force sensor. For consistency, we retained the grasp force measure in our analyses. The remaining metrics were still sufficient to show a significant main effect of *tactile feedback*.

A within-subjects ANOVA, with factors of *visual feedback condition *(light block/dark block), *tactile feedback condition *(with vibrotactile feedback/without vibrotactile feedback) and *phase *(phase one/phase two) revealed a significant main effect of *visual feedback condition *(*F*(3, 8) = 6.91, *p *= .013) and a significant main effect of *tactile feedback condition *(*F*(3, 8) = 7.51, *p *= .010). There was no significant main effect of *phase *(*F*(3, 8) = 1.56, *p *= .274), and there were no significant interactions (*F*(3, 8) ≤ 2.17, *p *≥ .169).

Post-hoc comparisons revealed that the cause of the effects was best explained with the *grasp score *measure (see Figure 6) As an additional analysis, we compared the *grasp score *measure for the various feedback conditions in the second phase of trials. In trials without visual feedback we found a significant effect of tactile feedback (*F*(1, 11) = 6.4, *p *= .028), but with visual feedback there was no significant effect of tactile feedback (*F*(1, 11) = 0.405, *p *= .538). We also found that without tactile feedback there was a significant effect of visual feedback (*F*(1, 11) = 9.27, *p *= .011), but with tactile feedback there was no significant effect of visual feedback (*F*(1, 11) = 0.231, *p *= .640). This suggests that, after training, either modality was sufficient to enable task performance (see *discussion*).

## Discussion

The purpose of our first experiment was to quantify the benefits of tactile feedback in an idealised grasping and lifting task. We used *grasp economy *as our measure of performance, a phenomenon known to depend on feedback and feedforward predictions (see *introduction*). It has previously been shown that two chronically deafferented patients were not significantly different from healthy matched controls at scaling grip force to different object weights [18].

A study to quantify the benefits of artificial feedback for force control also found no significant difference between feedback and no-feedback groups [26]. Consistent with these studies, we found no effect of tactile feedback condition, yet we found a highly significant effect of object weight, indicating economical grasps regardless of tactile feedback. A preliminary experiment had confirmed that our feedback system offered adequate bandwidth to subjects. We therefore suspected that, under the ideal conditions of experiment 1, subjects' ability to grasp economically was due to abundant sensory cues (from visual and auditory modalities).

Contrary to our hypothesis, in our second experiment subjects were still capable of differentiating object weights and applying appropriately economical grip forces when deprived of all sources of sensory feedback. We found no significant difference in grasp economy between two groups, one with vibrotactile feedback and one without, nor did we find a significant difference between the light and dark conditions. It has been previously shown in healthy humans that cutaneous feedback enables maintenance of the anticipatory components of grasping [18], but our results suggest that, under the idealised control conditions, force feedback was not necessary for this purpose. However, we did find a higher overall grip force in the absence of visual feedback, consistent with an increased safety-margin observed in feedback-deprived individuals [20]. Nevertheless, subjects still differentiated the two objects, which requires precise signal timing in order to set appropriate grasp forces. Since the objects were lifted multiple times, we concluded that subjects were able to learn an internal model in the absence of within-trial feedback. We posit that a *feedforward process *was playing a crucial role in the observed behaviour.

The results of our third experiment showed that when feedforward predictability was degraded, performance degraded too. However, with the addition of either visual or tactile feedback, performance was restored, providing evidence that feedback is required in the presence of feedforward uncertainty. Best performance was achieved in the presence of both sources of feedback, suggesting that visual and tactile cues play complementary roles in facilitating successful grasps in the presence of uncertainty.

In this study we used a vibrotactile feedback interface. Direct pressure-feedback devices [27] may offer a more natural sensation, and electrotactile feedback might provide greater spatial resolution [28] at the expense of safety. However, vibrotactile feedback systems are given credit for their low cost, size and weight and the simplicity and flexibility with which they can be used in sensory substitution applications [29]. For these practical reasons we developed a spatially-encoded vibrotactile feedback interface (similar to [30]). In pilot studies we have found that this method affords greater stimulus bandwidth than a single tactor providing frequency- or amplitude-encoded feedback, as well as reduced adaptation (data not shown). To make the argument that subjects were adequately trained to use the vibrotactile feedback we conducted an preliminary trial which revealed that subjects were immediately able to discriminate tactile stimuli, and it offered a sufficient perceptual range. Furthermore, subjects were able to utilise vibrotactile feedback to their advantage in the third experiment. It is possible that with considerably more training we may have observed a difference in performance between the vibrotactile group and non-vibrotactile group in experiment 2. However, this does not invalidate the finding that subjects could form economical grasps *regardless *of feedback under ideal experimental conditions.

It is likely that our observations were a result of the ideal control conditions we created. Since blocks of trials were in a predictable order and subjects performed multiple repeated trials per object, subjects could learn by trial-and-error. Furthermore, subjects were aware of a successful lift via feedback from their arm muscles as well as on-screen feedback at the end of each trial, allowing them to refine their judgements. Our work assumes that, by these processes, subjects can establish a feedforward prediction. This is defined as the ability to anticipate the forces they are exerting in the absence of *externally-arising *cues to that fact (see *introduction*). It is important to note that proprioceptive and tactile cues of the control signal are considered to be *internal *cues -- they provide no feedback of how the robotic hand is interacting with the environment. However, it should also be noted that, in contrast to our ideal controller, commercially available prostheses are typically controlled by noisy EMG signals and that prosthesis control methods often do not provide predictable force control. Our results indicate that predictable control can obviate the practical benefits of feedback. However, in the presence of unavoidable feedforward uncertainty the benefits of feedback are apparent.

In this study we induced random temporal delays when simulating feedforward uncertainty in experiment 3. Temporal uncertainty and temporal judgement impact many dexterous tasks, in both healthy humans and prosthesis wears. At the task-level one can expect unpredictable sensory and motor delays [31], such as when grasping objects of unknown size or shape, or when not paying full visual attention. Every motor action is undertaken in the presence of uncertainty [32], resulting in some degree of temporal error. Temporal uncertainty is also a considerable concern for prosthesis designers. Since EMG signals used to initiate and control prosthesis movement fluctuate as a function of sweat, movement, muscle fatigue and skin-conductivity [33] the most reliable EMG classifiers require 250-300 ms of sampling time before accurate classification can be made [34]. In the interest of responsiveness, controllability and expense, many commercially available prostheses use differential ("open/close") controllers to defer the problem of EMG signal reliability to the temporal domain. Our results reveal that temporal uncertainty can significantly impair performance, but these effects are reduced with appropriate feedback.

To our knowledge this research provides first demonstration of the existence of feedforward and feedback processes for an artificial limb. Our results support, and perhaps provide an explanation for, similar studies in the literature. A study that showed no significant prosthesis control improvements with vibrotactile feedback [26] could be explained by our finding of a strong feedforward contribution. The benefit of feedback in the presence of partial sensory deprivation [16] or with visual distractions [35] is supported by our finding of the role of feedback in the presence of uncertainty. Furthermore, we assert that our result is widely applicable to research into human perception and sensorimotor control. In line with studies involving deafferented [18,21] and anaesthetised patients [20], our work supports the computational view of sensorimotor learning under uncertainty [32].

We have shown quantitatively that tactile feedback can significantly improve performance in the presence of feedforward uncertainty. These results have important implications for the prosthetics field, and consequently we make three recommendations: (i) Prostheses should be designed to make control as predictable and repeatable as possible, to minimise feedforward uncertainty; (ii) Feedback should be provided to handle the inevitable uncertainty that will arise, and should be chosen to enable better feedforward learning (such as error-corrective feedback, or force-derivative feedback, described in [36]); and (iii) We should aim to exploit the different sources of noise between robotic and human systems: trade-offs in design, for example, allow temporal uncertainty to be transformed into spatial uncertainty. If we can minimise uncertainty in task-specific domains we may increase control reliability and considerably improve hand functionality.

This study raises a number of interesting possibilities for future work. We have presented here a robotic system that replaces the healthy sensorimotor system for the elementary task of object lifting, but what are the limits of this analogy? Amputees fitted with prostheses such as the one presented in this paper will not have the benefit of 'idealised control': real-world prostheses are controlled by EMG electrodes which, as previously discussed, add control uncertainty. Our results suggest that EMG control will result in diminished grasp economy that can be remedied either by improving the reliability of EMG measurement (reducing feedforward uncertainty) or through provision of a reliable limb-state feedback. Our robotic manipulandum also provides a viable platform to test this hypothesis. Multifunction prostheses of the future offer increased dexterity and functionality at the expense of additional feedforward and feedback demands (as discussed in [37]). Tasks involving dynamic or unstable loads, such as handwriting, or tying shoelaces, require the learning of much more complex internal models. It is not obvious how these models are acquired, nor how they depend on motor control or available feedback, yet they are key to the design of a system that needs to mimic human behaviour. We argue that our novel manipulandum is an ideal platform to study human sensorimotor processes as it allows the experimenter to access sensory and motor components that, in intact individuals, is either unethical or practically impossible.

Our results suggest that feedback should be chosen to complement the uncertainty in the control system. This does not mean, however, that by removing all uncertainty from the controller we will remove the necessity for feedback: a device which acts automatically and intelligently will surely reduce the number of grasping errors, but may not be accepted by the amputee as a natural extension of their nervous system. Vivid sensations of *embodiment *and prosthesis *ownership *can only be achieved through physiologically appropriate cutaneous feedback [15].

## Conclusions

We have presented here an original method to decouple the role of sensory and anticipatory components of human grasping. Using our novel manipulandum we have shown quantitatively that feedforward and feedback processes are co-dependent. This is the first demonstration of the existence of feedforward and feedback processes for an artificial limb, a phenomenon well characterised for the healthy nervous system, and is therefore an important step in understanding the human-machine interface.

By manipulating feedforward and feedback uncertainty we have shown that the seemingly trivial task of grasping and lifting objects employs non-trivial cognitive mechanisms. We might exploit this by designing prostheses with predictable feedforward controllers and feedback systems that allow users to correct for inevitable control uncertainty.

## Competing interests

The authors declare that they have no competing interests.

## Authors' contributions

IS contributed to all stages of this research (i.e. planning, implementation, conducting experiments and writing). IS conceived the concept of the novel manipulandum, designed and built the requisite vibrotactile feedback hardware and developed the software and firmware required for control of the i-LIMB hand. All stages were completed under the supervision of SV. Both authors read and approved the final manuscript.

## References

[B1] Shannon GF A myoelectrically-controlled prosthesis with sensory feedback Med Biol Eng Comput 1979 17 73 80 10.1007/BF02440956 312386

[B2] Scott RN Brittain RH Caldwell RR Cameron AB Dunfield VA Sensory-feedback system compatible with myoelectric control Med Biol Eng Comput 1980 18 65 69 10.1007/BF02442481 7382591

[B3] Riso RR Ignagni AR Keith MW Cognitive feedback for use with FES upper extremity neuroprostheses IEEE Trans Biomed Eng 1991 38 29 38 10.1109/10.68206 2026429

[B4] Edin BB Ascari L Beccai L Roccella S Cabibihan JJ Carrozza MC Bio-inspired sensorization of a biomechatronic robot hand for the grasp-and-lift task Brain Res Bull 2008 75 6 785 795 10.1016/j.brainresbull.2008.01.017 18394525

[B5] Ascari L Bertocchi U Corradi P Laschi C Dario P Bio-inspired grasp control in a robotic hand with massive sensorial input Biological cybernetics 2009 100 2 109 128 10.1007/s00422-008-0279-0 19066937

[B6] Kuiken TA Dumanian GA Lipschutz RD Miller LA Stubblefield KA The use of targeted muscle reinnervation for improved myoelectric prosthesis control in a bilateral shoulder disarticulation amputee Prosthet Orthot Int 2004 28 3 245 253 15658637 10.3109/03093640409167756

[B7] Dhillon GS Horch KW Direct neural sensory feedback and control of a prosthetic arm IEEE Trans Neural Syst Rehabil Eng 2005 13 4 468 472 10.1109/TNSRE.2005.856072 16425828

[B8] Miller LA Lipschutz RD Stubblefield KA Lock BA Huang H Williams TW Weir RF Kuiken TA Control of a six degree of freedom prosthetic arm after targeted muscle reinnervation surgery Arch Phys Med Rehabil 2008 89 11 2057 2065 10.1016/j.apmr.2008.05.016 18996233 PMC3032984

[B9] Zhou P Lowery MM Englehart KB Huang H Li G Hargrove L Dewald JPA Kuiken TA Decoding a new neural machine interface for control of artificial limbs J Neurophysiol 2007 98 5 2974 2982 10.1152/jn.00178.2007 17728391

[B10] Carrozza MC Cappiello G Micera S Edin BB Beccai L Cipriani C Design of a cybernetic hand for perception and action Biol Cybern 2006 95 6 629 644 10.1007/s00422-006-0124-2 17149592 PMC2779386

[B11] Iyengar V Santos MJ Ko M Aruin AS Grip force control in individuals with multiple sclerosis Neurorehabil Neural Repair 2009 23 8 855 861 10.1177/1545968309338194 19531607

[B12] Jiang L Cutkosky MR Ruutiainen J Raisamo R Using Haptic Feedback to Improve Grasp Force Control in Multiple Sclerosis Patients IEEE Transactions on Robotics 2009 25 3 593 601

[B13] Cipriani C Antfolk C Balkenius C Ros'en B Lundborg G Carrozza MC Sebelius F A novel concept for a prosthetic hand with a bidirectional interface: a feasibility study IEEE Trans Biomed Eng 2009 56 11 Pt 2 2739 2743 19758852 10.1109/TBME.2009.2031242

[B14] Panarese A Edin BB Vecchi F Carrozza MC Johansson RS Humans can integrate force feedback to toes in their sensorimotor control of a robotic hand IEEE Trans Neural Syst Rehabil Eng 2009 17 6 560 567 19457753 10.1109/TNSRE.2009.2021689

[B15] Marasco PD Kim K Colgate JE Peshkin MA Kuiken TA Robotic touch shifts perception of embodiment to a prosthesis in targeted reinnervation amputees Brain 2011 10.1093/brain/awq361 PMC3044830 21252109

[B16] Zafar M Doren CLV Effectiveness of supplemental grasp-force feedback in the presence of vision Med Biol Eng Comput 2000 38 3 267 274 10.1007/BF02347046 10912342

[B17] Jordan MI Wolpert DM Computational Motor Control The Cognitive Neurosciences 1999 601

[B18] Hermsdörfer J Elias Z Cole JD Quaney BM Nowak DA Preserved and impaired aspects of feed-forward grip force control after chronic somatosensory deafferentation Neurorehabil Neural Repair 2008 22 4 374 384 18223241 10.1177/1545968307311103

[B19] Flanagan JR Wing AM The role of internal models in motion planning and control: evidence from grip force adjustments during movements of hand-held loads J Neurosci 1997 17 4 1519 1528 9006993 10.1523/JNEUROSCI.17-04-01519.1997 PMC6793733

[B20] Augurelle AS Smith AM Lejeune T Thonnard JL Importance of cutaneous feedback in maintaining a secure grip during manipulation of hand-held objects J Neurophysiol 2003 89 2 665 671 12574444 10.1152/jn.00249.2002

[B21] Hermsdörfer J Hagl E Nowak DA Deficits of anticipatory grip force control after damage to peripheral and central sensorimotor systems Hum Mov Sci 2004 23 5 643 662 10.1016/j.humov.2004.10.005 15589626

[B22] Johansson RS Westling G Roles of glabrous skin receptors and sensorimotor memory in automatic control of precision grip when lifting rougher or more slippery objects Exp Brain Res 1984 56 3 550 564 6499981 10.1007/BF00237997

[B23] Otr OVDNV Reinders-Messelink HA Bongers RM Bouwsema H Van Der Sluis CK The i-LIMB hand and the DMC plus hand compared: a case report Prosthetics and orthotics international 2010 34 2 216 220 10.3109/03093641003767207 20470060

[B24] Humbert SD Snyder SA Grill WM Evaluation of command algorithms for control of upper-extremity neural prostheses IEEE Trans Neural Syst Rehabil Eng 2002 10 2 94 101 10.1109/TNSRE.2002.1031977 12236452

[B25] Westling G Johansson RS Factors influencing the force control during precision grip Exp Brain Res 1984 53 2 277 284 6705863 10.1007/BF00238156

[B26] Chatterjee A Chaubey P Martin J Thakor NV Quantifying Prosthesis Control Improvements Using a Vibrotactile Representation of Grip Force Proc IEEE Region 5 Conference 2008 1 5

[B27] Patterson PE Katz JA Design and evaluation of a sensory feedback system that provides grasping pressure in a myoelectric hand J Rehabil Res Dev 1992 29 1 8 1740774 10.1682/jrrd.1992.01.0001

[B28] Kaczmarek KA Webster JG Bach-y Rita P Tompkins WJ Electrotactile and vibrotactile displays for sensory substitution systems IEEE Transactions on Biomedical Engineering 1991 38 1 16 10.1109/10.68204 2026426

[B29] Alahakone AU Senanayake SMNA Vibrotactile feedback systems: Current trends in rehabilitation, sports and information display Proc IEEE/ASME International Conference on Advanced Intelligent Mechatronics AIM 2009 2009 1148 1153

[B30] Cholewiak RW Collins AA The generation of vibrotactile patterns on a linear array: influences of body site, time, and presentation mode Percept Psychophys 2000 62 6 1220 1235 10.3758/BF03212124 11019618

[B31] Kennedy JS Buehner MJ Rushton SK Adaptation to sensory-motor temporal misalignment: instrumental or perceptual learning? Q J Exp Psychol (Colchester) 2009 62 3 453 469 10.1080/17470210801985235 18609410

[B32] Bays PM Wolpert DM Computational principles of sensorimotor control that minimize uncertainty and variability J Physiol 2007 578 Pt 2 387 396 17008369 10.1113/jphysiol.2006.120121 PMC2075158

[B33] Duchêne J Goubel F Surface electromyogram during voluntary contraction: processing tools and relation to physiological events Crit Rev Biomed Eng 1993 21 4 313 397 8243094

[B34] Lorrain T Jiang N Farina D Surface EMG classification during dynamic contractions for multifunction transradial prostheses Conf Proc IEEE Eng Med Biol Soc 2010 1 2766 2769 10.1109/IEMBS.2010.5626587 21096217

[B35] Cincotti F Kauhanen L Aloise F Palomäki T Caporusso N Jylänki P Mattia D Babiloni F Vanacker G Nuttin M Marciani MG Millán JDR Vibrotactile feedback for brain-computer interface operation Comput Intell Neurosci 2007 48937 10.1155/2007/48937 PMC2267023 18354734

[B36] Engeberg ED Meek S Improved Grasp Group Force Sensitivity for Prosthetic Hands Through Force Derivative Feedback IEEE transactions on bio-medical engineering 2008 10.1109/TBME.2007.912675 18270026

[B37] Dosen S Cipriani C Kostić M Controzzi M Carrozza MC Popović DB Cognitive vision system for control of dexterous prosthetic hands: experimental evaluation Journal of neuroengineering and rehabilitation 2010 7 42+ 10.1186/1743-0003-7-42 20731834 PMC2940869

